# Engineering immune niches: biochemical, mechanical, and spatial design principles for translational hydrogels

**DOI:** 10.1007/s44258-026-00087-5

**Published:** 2026-06-22

**Authors:** Robert Hincapie, Oriana Marrone Mantovani, José McFaline-Figueroa, Santiago Correa

**Affiliations:** 1 Department of Biomedical Engineering, Columbia University, New York, NY 10027 USA; 2 Herbert Irving Comprehensive Cancer Center, Columbia University, New York, NY 10032 USA; 3 Irving Institute for Cancer Dynamics, Columbia University, New York, NY 10027 USA

**Keywords:** Immune niche, Immunoengineering, Hydrogels, Scaffolds, Spatial transcriptomics, Vaccination, Wound healing, Immune–stromal interactions

## Abstract

Leveraging advanced materials is an increasingly important direction in immune engineering, which aims to reprogram immune responses to treat disease. Once implanted, biomaterials rapidly become sites where immune cells assemble, interact, and collectively shape material performance. These organized multicellular environments, or immune niches, are linked to outcomes ranging from tissue regeneration to durable immune memory, yet the principles governing their formation and function remain poorly understood. In this Mini-Review, we examine how engineered translational hydrogels act both as modulators of immune niches and as tractable in vivo model systems for studying them. We highlight how biochemical cues such as antigens, adjuvants, cytokines, and chemokines control which cells enter a niche and how they become activated, and how biophysical properties including stiffness, viscoelasticity, porosity, and degradability influence cellular access, motility, and phenotype. Adhesion motifs are discussed as a hybrid class of signals that couple biochemical recognition to mechanical force transmission. We also describe how emerging spatial and multi-omic technologies are beginning to reveal the architecture and communication networks that define hydrogel-associated niches. Future progress will require close collaboration between materials scientists, immunologists, and computational biologists to establish the design principles needed to engineer immune niches that improve therapeutic outcomes.

**Graphical Abstract:**

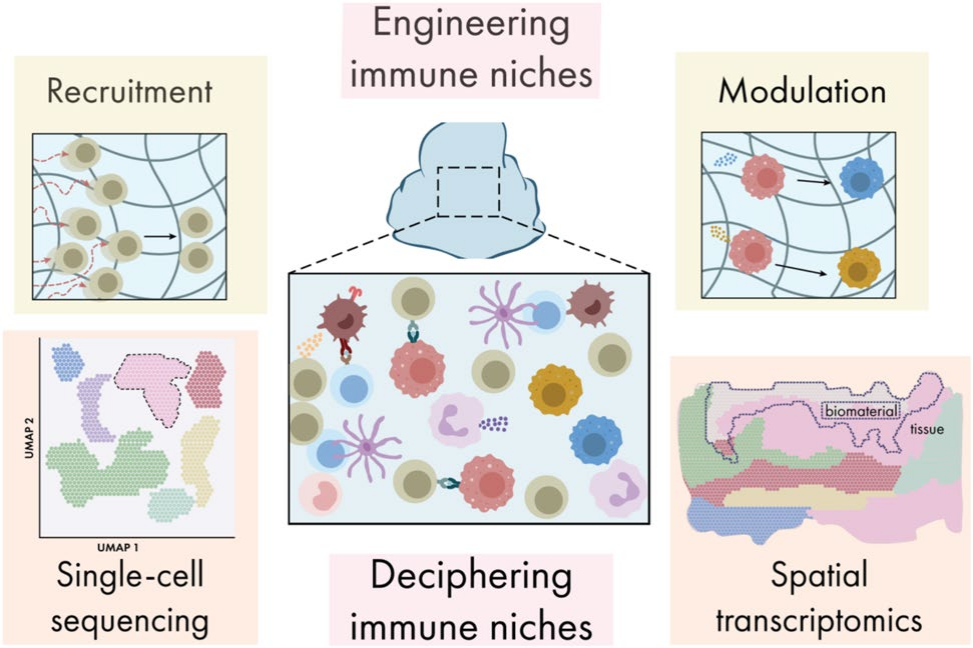

## Highlights

- Implanted biomaterials recruit cells that form multicellular communities, immune niches, shaping functional outcomes.
- Biochemical cargo, matrix mechanics, and adhesion cues shape which cells are recruited and how they behave in niches.
- Single-cell and spatial technologies reveal how immune niches are organized and determine functional outcomes.
- Immune niche engineering requires collaboration across materials science, immunology, and computational biology.

## Defining and investigating immune niches

The immune system is generally broken down into discrete cellular elements (*e.g., *the T cell, the B cell), each responsible for specific effector functions [1]. As our understanding has deepened, immunologists have also come to define the systems-level cellular networks that give rise to nuanced immune responses (*e.g.,*the multicellular interactions that give rise to a Th2 adaptive response) [2]. An important but comparatively understudied frontier in this regard is the immune system’s capacity to organize into spatially distinct multicellular communities, or immune niches [3, 4], in response to diverse stimuli.

Immune niches locally regulate immune activity, establishing microenvironments that can shape the nature and outcome of an immune response. For example, distinctive immune niches emerge during injury [5], vaccination [6, 7], and tumorigenesis [8], where they are closely linked to clinical outcomes: some promote tissue repair [9, 10], durable immunity [11, 12], and therapeutic responsiveness [13, 14], whereas others contribute to chronic wounds [15], suboptimal vaccine efficacy [16], or resistance to immunotherapy [17, 18]. Similarly, specialized immune niches can form in response to implanted biomaterials, such as orthopedic implants [19], pacemakers [20, 21], and hydrogels [22], where these niches can hinder or enhance the function of these biomaterials [23, 24]. In certain instances, immune niches with similar cellular compositions may be associated with divergent outcomes [25]. For example, the presence of tertiary lymphoid structures in cancers have been associated with both favorable [13, 14, 25] and unfavorable [17, 18] clinical outcomes, indicating that cell composition alone is not predictive of function, suggestive that other properties (*e.g.,* spatial organization) may be key to understanding the function of these niches. Across these diverse contexts, it is clear that the immune niche is a critical determinant of homeostasis, yet the mechanisms that govern its formation remain difficult to dissect in vivo.

Engineered biomaterials provide an opportunity to recreate and investigate these complex environments under controlled conditions. Hydrogels, in particular, have emerged as powerful tools for modeling and manipulating immune niches [26]. These materials can be engineered for diverse biomedical applications, including regeneration [27], tissue engineering [28], disease monitoring [29, 30], and drug [31], vaccine [22], and cell [32, 33] delivery. When injected or implanted, hydrogels can recruit and interact with infiltrating immune cells while presenting defined physical and biochemical cues that shape cell fate and behavior. Because injectable hydrogels are minimally invasive, tunable, and translationally relevant [26], these materials offer a tractable experimental system through which to study how immune niches form and how they regulate immunity.

This Mini-Review focuses on in vivo immune niches formed in response to injectable and implantable hydrogel systems, which offer precise control over biochemical signaling and mechanical properties within a physiologically relevant context. We discuss implantable fibrous extracellular matrix (ECM) scaffolds in the context of wound healing, and to highlight instances in which spatial transcriptomics (ST) has been used to profile the immune niche. Other classes of implantable biomaterials (*e.g., *solid porous matrices [34], particulate carriers [35], and granular or microporous gels [36]) provide complementary strategies but are beyond the scope of this discussion. Likewise, ex vivo manipulation of immune cells for therapeutic or modeling purposes, such as in organoids, organs-on-chips, or cellular immunotherapy platforms, is not covered here and has been reviewed elsewhere [37–39].

**Fig. 1:**
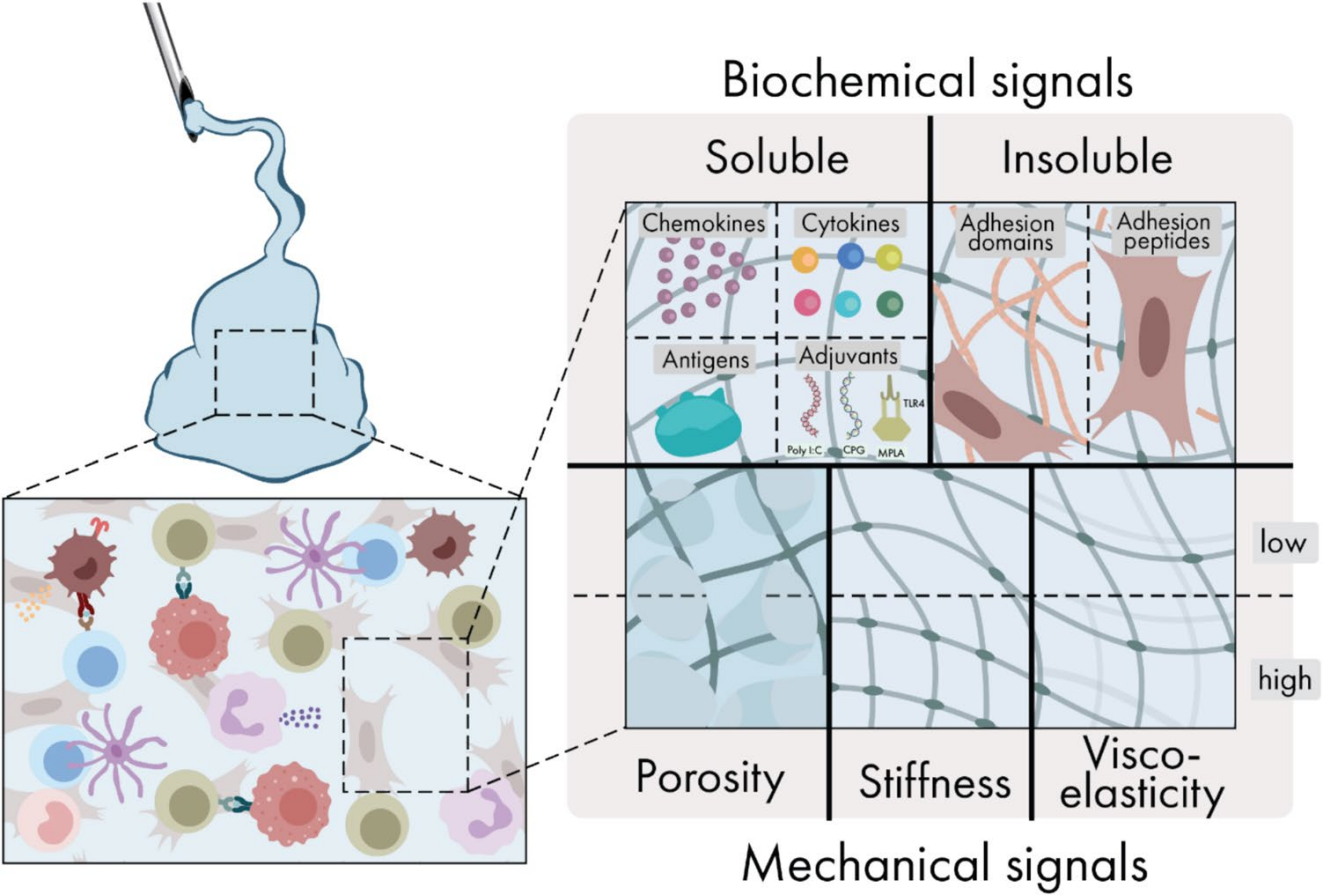
Injectable hydrogels as cell-instructive immune niches. Soluble signals (top, right) including chemokines, cytokines, antigens, and adjuvants can be encapsulated and released to recruit and guide immune cell behavior. Extracellular matrix-associated adhesion domains and adhesion peptides (e.g., RGD, GFOGER) can likewise be entrapped or immobilized to provide cues that mediate cell adhesion and can shape cell differentiation. Mechanical properties of hydrogels, including porosity, stiffness, and viscoelasticity, can be independently tuned and these features likewise drive cellular behavior

Engineered biomaterials, and particularly hydrogels, can be used to create and dissect immune niches in vivo (Fig. 1). These systems provide a means to uncover the design rules needed to rationally engineer immune niches that enhance biomaterial function and improve clinical outcomes. We focus on two primary engineering axes that shape niche formation: (i) biochemical control through the delivery of antigens, adjuvants, cytokines, chemokines, and other molecular signals that influence immune cell recruitment and activation; and (ii) biophysical control through properties such as stiffness, viscoelasticity, porosity, and degradability that regulate cellular access, motility, and mechanotransduction. Adhesion motifs are discussed as a special case that bridges these two axes by coupling molecular recognition to force transmission through the cytoskeleton.

We also consider recent advances in spatial and multi-omic analyses that reveal how immune niches are organized and evolve over time. Because the techniques required to characterize these processes often extend beyond those typically used in biomaterials research, we provide brief overviews of Key Techniques throughout the Mini-Review to introduce important analytical tools applied in the case studies discussed.

## Engineering the immune niche through biochemical and biomechanical cues

Engineering the immune niche requires understanding how material design parameters influence two interconnected processes: the recruitment of immune cells into a local microenvironment and their subsequent modulation once they arrive. Together, these processes determine the composition and functional state of the niche. Recruitment can occur *directly*, when immune cells respond to biochemical or physical cues presented by a material, or *indirectly*, when resident or ‘first responder’ cells sense the material and secrete their own molecules to recruit a subsequent influx of cells. Modulation encompasses the phenotypic changes that follow these interactions, leading to outcomes that range from inflammation to tolerance or regeneration. Dissecting how biochemical and biomechanical cues govern these steps provides the basis for rationally engineering immune niches.

In the following subsections, we examine how biochemical signals shape immune-cell recruitment and activation (Section “Biochemical strategies to shape immune niche composition and function”), how mechanical properties regulate cell migration and mechanotransduction (Section “Mechanical properties regulate immune niche fate”), and how adhesion motifs bridge these two axes through chemo-mechanical signaling (Section “Integrin-mediated adhesion as a chemo-mechanical regulator of niche dynamics”).

### Biochemical strategies to shape immune niche composition and function

Biochemical cues govern both the recruitment and the modulation of immune cells within a niche. Chemokines are classic examples, as they direct recruitment through chemotaxis, guiding immune-cell infiltration into a biomaterial or tissue [40]. Yet these molecules can also drive non-motility cellular functions that are sometimes overlooked. Many chemokines alter cell phenotype, polarizing cells toward inflammatory or regulatory states that, in turn, influence the secretion of additional molecules [40, 41]. In this way, chemokines can create feedback loops that further recruitment by modulating the behavior of the cells they initially attract. Likewise, cytokines are often considered functional cues that primarily drive modulation, such as activating macrophages [42], skewing T-cell differentiation [43], or promoting tissue remodeling [44, 45]. However, cytokines can also affect migration and retention by altering the chemokine profile or adhesion properties of responding cells [46]. This ‘domino effect’ is a common aspect to any biochemical cargo or biomaterial that perturbs immune function.

Beyond soluble factors, extracellular vesicles (EVs) are emerging as an additional class of biochemical signal; secreted by most cells, EVs carry a variety of cell-associated signaling molecules (cytokines, glycans, lipids, miRNAs, and others) [47] and have diverse immunomodulatory functions. EVs have recently been incorporated into biomaterials to promote tissue regeneration [48], and as platforms for tumor immunotherapy [49], or vaccine release [50]. In particular, there has been considerable enthusiasm for EVs as an alternative to stem cell therapy, as EVs obtained from stem cells appear to recapitulate many of the pro-regenerative benefits seen with cell therapies [51, 52]. Relative to chemokines and cytokines, which are well-defined in terms of their composition and function, EVs and other cell-derived nanovesicles are highly heterogeneous in terms of their origin, size, composition, and overall function [53]. This complexity also hinders characterization and standardization of EVs, although systemic guidelines and recommendations for EV analysis are routinely updated by the International Society for Extracellular Vesicles [47].

These overlapping roles illustrate that recruitment and modulation are deeply intertwined processes. Manipulating one almost inevitably influences the other, meaning that the selection of a biochemical cue is not only a choice of which cell to attract, but also of what state to induce and how that state feeds back into the evolving composition of the niche.

Hydrogels offer a practical framework to explore and control these biochemical dynamics. Molecular cues can be incorporated through encapsulation [54], affinity tethering [55, 56], or covalent conjugation [57] to achieve defined concentrations and release kinetics. These strategies may be used in combination to control staged release of biomolecules from hydrogels [56]. This level of control is particularly advantageous in recapitulating the complex biochemical environment of native tissues, where cues are presented not only at specific times but also at defined locations and often in gradient-forming patterns. Anseth and coworkers, for instance, pioneered the use of photochemistry to covalently pattern peptide gradients within hydrogels [58]. More broadly, photochemical customization of hydrogels enables spatial patterning of biochemical cues and control over matrix properties, and has been recently reviewed elsewhere [59, 60]. We do not review these incorporation methods in detail here (see Refs. [61, 62] for comprehensive overviews), but highlight that cue identity, dose, and temporal presentation are key axes governing immune-niche outcomes. Each of these variables can shift the balance between recruitment and modulation, amplifying or dampening immune activation in ways that remain difficult to predict because many signaling molecules are pleiotropic and context dependent [46, 63]. Achieving rational control over biochemical signaling within hydrogels therefore remains a central challenge for immune-niche engineering.

**Fig. 2:**
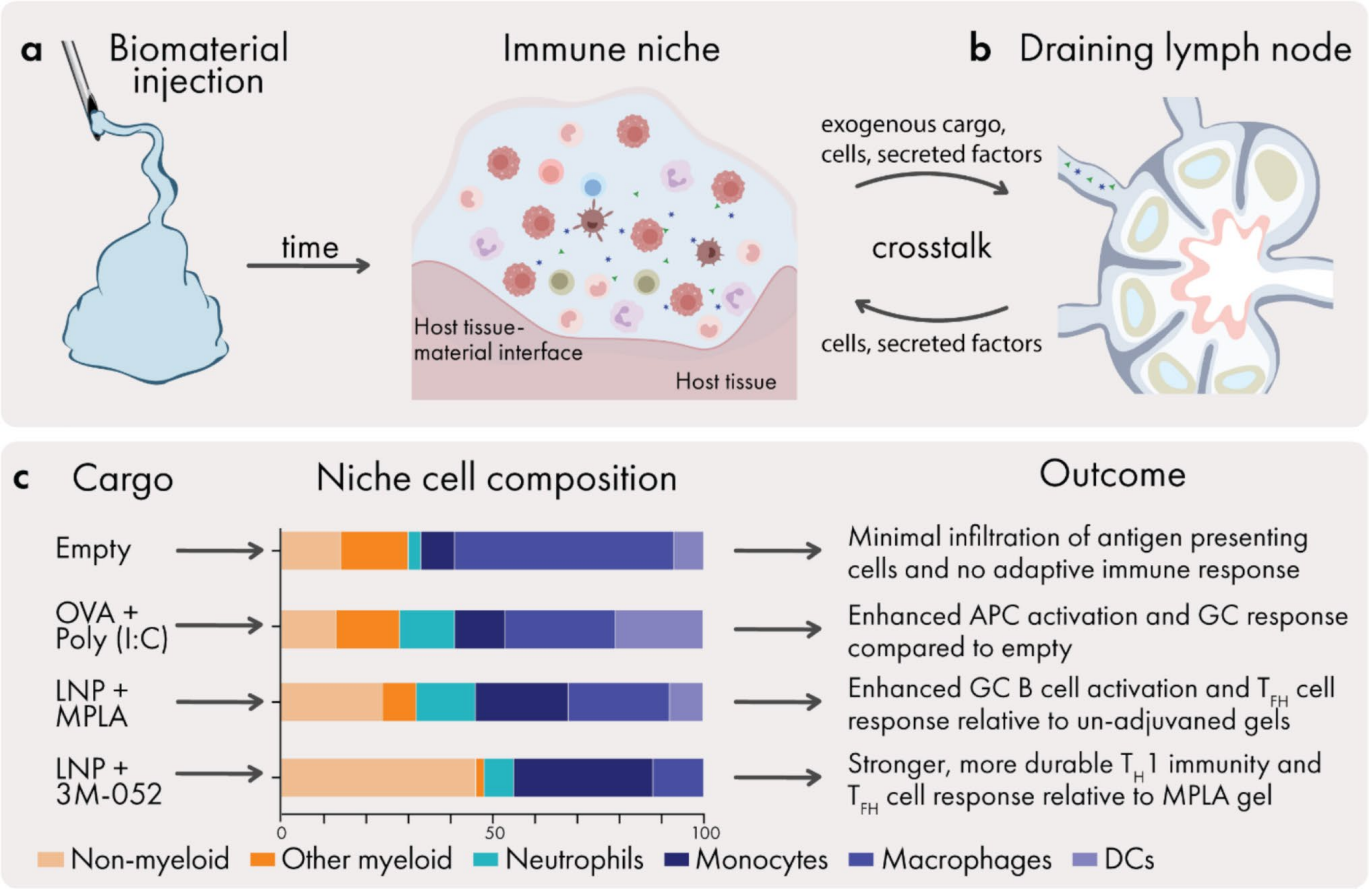
Hydrogel cargo shapes the immune niche. **a** Injectable hydrogels containing encapsulated antigens (e.g., OVA, SARS-CoV-2 spike protein mRNA) and adjuvants (e.g., Poly(I:C), monophosphoryl lipid A (MPLA)) recruit immune cells to the site of injection. **b** Afferent vessels carry factors released from the hydrogel, or from cells recruited into the hydrogel, to draining lymph nodes where they are taken up and processed by resident immune cells. Antigen presenting cells, activated T cells, chemokines, and cytokines exit lymph nodes from efferent vessels, where they can enter circulation or be directed towards a hydrogel niche. **c** Hydrogel encapsulated cargo helps determine the cell composition and the functional outcomes of the immune niche. Relative to other materials, empty hydrogels lacking antigen and adjuvant were found to have minimal infiltration of antigen presenting cells. The prolonged release of ovalbumin and the nucleic acid analog Poly(I:C) by hydrogels encapsulating these agents led to enhanced recruitment of antigen presenting cells (DCs and macrophages) in hydrogels and longer-lived germinal centers (GCs) in draining lymph nodes. Likewise, the encapsulation of glycolipid or small molecule adjuvants (MPLA and 3 M-052, respectively) alongside mRNA/LNP vaccines improved the quality of cell help by T follicular helper cells (T_FH_) and the frequency of antigen-specific germinal center B cells in draining lymph nodes relative to un-adjuvanted LNP hydrogels

A clear illustration of how cargo identity shapes the immune niche comes from the work of Appel and coworkers using polymer–nanoparticle (PNP) hydrogels as a platform to study subunit vaccine delivery (Fig. 2) [22]. In vaccines, adjuvants are molecular cues that activate innate immune pathways and license antigen-presenting cells to initiate robust adaptive responses. Appel and coworkers encapsulated the model antigen ovalbumin together with the toll-like receptor (TLR3) agonist Poly(I:C) in PNP hydrogels, creating a local inflammatory niche at the injection site. In vivo, Poly(I:C) dramatically altered the niche composition compared to empty gels, increasing the total number of infiltrating cells by nearly five-fold and enriching the niche with monocytes, macrophages, and migratory dendritic cell (DC) subsets that are capable of priming T cells.

This work proposed that dendritic cells recruited into the gel acquire antigen and become activated within this niche before migrating to the draining lymph node, where they sustain germinal center activity. Directly confirming this trafficking pathway remains challenging because current experimental designs cannot distinguish dendritic cells that interacted with the gel depot from those activated elsewhere. Methods developed by Mooney and colleagues, which use metabolically incorporated azido-sugars to label cells that contact a biomaterial [64], may eventually allow such fate mapping in future work.

Even without direct cell tracking, several observations are consistent with the proposed pathway: gel-based delivery of Ovalbumin and Poly(I:C) increased germinal center B-cell frequencies in the draining lymph node, produced higher and more durable serum antibody titers, and generated antibodies with affinities approximately three orders of magnitude greater than those elicited by bolus vaccination. Together, these findings show that encapsulating biochemical cargo within a hydrogel reprograms the local immune niche, and that these local changes propagate to changes in downstream lymphoid tissues that influence the quality, magnitude, and durability of the adaptive response. In this way, the immune niche established within a biomaterial is a plastic and intentionally tunable environment whose composition and function can be directed through adjuvant selection.

To extend this concept of niche plasticity, Meany et al. evaluated how incorporating clinically relevant mRNA/lipid nanoparticle (LNP) vaccines into PNP hydrogels, with or without defined adjuvants, alters both the local inflammatory niche and downstream immune responses [24]. The authors’ motivation stemmed from a well-recognized limitation of conventional mRNA vaccines: following bolus injection, LNPs rapidly diffuse, transfect a mixture of professional and non-professional antigen-presenting cells, and are degraded within 24–72 h, limiting both antigen availability and control over the early microenvironment where immune priming begins. Encapsulating LNPs in PNP hydrogels was hypothesized to (i) target LNPs to recruited antigen presenting cells (APCs) within a confined niche, (ii) prolong the availability of antigen, and (iii) enable modular inclusion of molecular adjuvants, here monophosphoryl lipid A (MPLA, a TLR4 agonist) and 3 M-052 (TLR7/8 agonist). Readers seeking an overview of the functional roles of these TLR pathways are referred to authoritative reviews on innate immunology [65, 66]. This approach allowed the investigators to compare how different adjuvants, delivered in an identical hydrogel framework, drive distinct niche environments and thereby modulate vaccine efficacy.

Compared to LNP-only hydrogels, formulations containing adjuvants reshaped the local inflammatory niche in distinct ways (Fig. 2c). Both MPLA and 3 M-052 produced a marked expansion of the myeloid compartment, driven primarily by increased recruitment of neutrophils and monocytes. However, classical dendritic cells were consistently more abundant in MPLA-containing hydrogels at both early and later time points, whereas 3 M-052–containing hydrogels showed fewer classical DCs but a more pronounced influx of inflammatory monocytes and neutrophils, and at later stages, an enrichment of NK cells. Because DC recruitment is typically viewed as a hallmark of effective vaccine design [67, 68], one might expect the MPLA hydrogels to outperform the 3 M-052 formulation. Instead, the opposite was observed: 3 M-052–adjuvanted hydrogels elicited stronger and more durable germinal center reactions, greater T follicular helper cell responses, enhanced antibody titers and durability, broader variant recognition, and production of IgG2c antibodies consistent with Th1 immunity. Evidently, it is not yet clear how to map differences in immune niche composition and modulation over time to biological outcomes.

**Table:** 

**Key Technique #1: Flow Cytometry.** This single-cell technique enables users to profile the composition and phenotypes of cells within a particular microenvironment. In a typical experiment, combinations of fluorescently-labeled antibodies, nanobodies, or other ligands are used to bind extracellular receptors or intracellular targets and identify cells on the basis of marker expression. More advanced techniques include permeabilization of cells to probe intracellular markers, such as in intracellular cytokine staining (ICS) assays, which employ Golgi-blocking agents to arrest cytokine secretion and allow intracellular accumulation of cytokines for detection, enabling functional characterization of cytokine-producing cell populations [69]. In-depth phenotyping allows users to maximize the information that can be obtained from an experiment: techniques such as mass cytometry [70] and spectral flow cytometry allows for panels containing 20–50 parameters [71–73], enabling users to more easily and comprehensively profile immune cells and their activation markers. Traditional flow cytometry workflows are invaluable, but sacrifice spatial information and insights into cell-cell interactions. A recently described workflow, Interact-omics, overcomes this limitation, allowing high-throughput quantitation of interacting cells (from multiplets) using standard flow cytometry data and a software toolkit [74].	

These observations reinforce that simple metrics such as DC frequency within the immune niche are insufficient for interpreting or predicting vaccine outcomes. One possibility is that dendritic cells accumulating in MPLA hydrogels may actually be evidence of impaired DC egress to lymph nodes, whereas the lower DC abundance in 3 M-052 hydrogels could reflect more rapid activation and egress. Another possibility is that conventional flow cytometry, which captures only a limited set of surface markers, cannot resolve the functional diversity within the recruited myeloid compartment. Cells with similar marker profiles may differ in cytokine production, antigen processing, migratory behavior, or TLR responsiveness, meaning that the subsets most relevant to vaccine efficacy remain indistinguishable with standard flow cytometry-based phenotyping [75, 76]. Higher-dimensional approaches such as single-cell transcriptomics or spatial profiling will likely be necessary to identify the specific myeloid states within the niche that drive superior lymph node responses and vaccine performance.

These studies also emphasize the importance of characterizing the niche over time, though this requires terminal experiments and is thus both costly and time-consuming. Because organ-on-chip models enable controlled but dynamic multi-cellular cultures, they may better support time-resolved mechanistic characterization of how niche dynamics shape functional outcomes. This question could be further addressed in vivo using approaches that enable real-time cell tracking. For example, migration and spatial localization of adoptively transferred fluorescent or bioluminescent cells can be tracked in vivo using optical techniques such as intravital imaging or in vivo imaging system (IVIS). The development of strategies that enable real-time monitoring of the immune niche will enable to more easily probe the formulation design space.

Together, these studies illustrate both the promise and the complexity of using biochemical cues to engineer immune niches in vivo. Changing the molecular cargo within a hydrogel can reshape the local myeloid milieu, alter APC activation and trafficking, and ultimately reprogram germinal center dynamics and antibody responses. Yet the biochemical design space is enormous: dozens of chemokines, nearly a hundred cytokines, multiple pattern-recognition receptor families, and numerous lectin pathways can all be engaged, each with pleiotropic and context-dependent effects. Only a small number of in vivo studies compare release, cell recruitment, or therapeutic efficacy of these signals when delivered from biomaterials [77–79], leaving much of this landscape unexplored. Moving forward, defining how specific biochemical inputs shape niche composition and immune outcome will likely require higher-dimensional phenotyping together with more systematic screening strategies, potentially aided by complementary platforms such as organ-on-chip systems that can sample broader design spaces than are currently feasible in vivo.

### Mechanical properties regulate immune niche fate

Much like how chemokines and cytokines provide biochemical information to cells, mechanical cues supply a parallel stream of signals that shape how immune cells move, what phenotypes they adopt, and how they coordinate within a biomaterial [80, 81]. Through mechanosensing, cells detect forces, stiffness, deformation, and other physical features of their surroundings; through mechanotransduction, they convert these cues into intracellular biochemical programs that alter behavior and cell fate [80]. Several types of mechanical input can be sensed: the stiffness or elasticity of a matrix [82], which determines the resistance a cell encounters as it spreads or contracts; tension and compressive forces [81], which are transmitted through integrins and the actin cytoskeleton; viscoelasticity [83], which governs how quickly a material relaxes under deformation; and porosity [84], which constrains infiltration and scaffold remodeling. A related concept is hydrogel architecture and morphology, which determine interconnectivity and complexity of the network [85, 86]. In combination, these physical properties form the mechanical information that immune cells interpret as they enter an environment. Understanding how these signals are sensed and integrated is therefore essential for designing niche environments that guide immune responses in predictable ways. For more information regarding mechanisms underlying mechanical modulation of immune cells, readers are referred to the following excellent reviews [80, 81, 83].

Hydrogels offer several practical strategies for tuning the mechanical cues that shape immune-cell behavior [26, 87]. Stiffness can be adjusted by changing polymer concentration, crosslink density, or crosslink chemistry, each of which alters how strongly the network resists deformation [88, 89]. Viscoelasticity, which reflects how a material dissipates rather than stores mechanical energy, can be modulated in several ways, for example by controlling porosity [90], crosslinking exchange rates [91, 92], and chain association strength [93]. Combinations of these parameters (e.g., polymer chain length and crosslinking density) [94] can be tuned together to decouple scaffold viscoelasticity from overall stiffness.

Using this strategy, Chaudhuri and colleagues have demonstrated that matrix viscoelasticity regulates the morphology, migration, and phenotypes of stem cells [95], fibroblasts [96], and cancerous cells [97]. In certain networks, small molecule crosslinkers can instead be used to modulate viscoelasticity, but not stiffness. Adu-Berchie and co-authors used the latter approach to modulate T cell expression of memory and activation markers as a function of hydrogel viscoelasticity [98]. Myeloid cells are also sensitive to viscoelasticity; THP-1-derived macrophages adopt more rounded and compact morphologies when cultured on rapidly stress-relaxing hydrogels, consistent with altered cytoskeletal organization in these materials [99].

Topological features including roughness and porosity are also key design elements to consider in cultivating an immune niche. Surface roughness and topography are known to impact cell migration and activation, as well as protein adsorption which can itself influence these factors [100]. Macrophages are particularly responsive to surface roughness of implants, which can be tailored to mitigate inflammation and fibrosis [101]. Porosity can be tuned by changing crosslink density, which controls the molecular mesh size, or by creating macroporous architectures, such as cryogels [102] or microporous annealed (MAP) gels [36], that permit immune-cell infiltration even when the underlying mesh is small [103]. Microparticle-based systems, including MAP or granular microgel systems, are comprised of hydrogel microparticles that are crosslinked or physically jammed together. These microgel systems have high degrees of porosity and pore interconnectivity that promote cell infiltration and support matrix remodeling by cells [86, 104].

Degradability also influences the mechanical niche because enzymatic or hydrolytic breakdown of the network increases effective pore size, reduces stiffness, accelerates stress relaxation, and enables cells to remodel or escape the material [105, 106]. Degradability can also be leveraged to control the temporal release of biochemical cues from hydrogels, which is important for recapitulating the dynamic environment of biological tissues and immune responses. For example, Kuan and colleagues designed a bilayer alginate hydrogel with orthogonal crosslinking strategies and distinct degradation profiles, to enable the staged release of IL-10 and angiogenic growth factors [107]. Degradation is also critical in navigating the fine line between immune stimulation and the activation of the foreign body response, which activates processes associated with fibrosis and ‘quarantining’ of a biomaterial [108].

Mechanical cues from the extracellular matrix are highly dynamic in tissue development, wound healing, and disease, and thus temporal control over material properties is critical to recapitulate biological signaling [86, 109]. The mechanical properties of hydrogels can be spatiotemporally controlled using dynamic materials that respond to user-defined cues, such as light [59, 60]. For example, hydrogel networks can be photochemically transformed to induce mechanical softening (by bond breaking reactions) [110], stiffening (by bond forming reactions), or increases in viscoelasticity (via bond rearrangement) [111]. Ultimately, the ability to trigger mechanical shifts in materials could provide new experimental systems to better understand structure–function properties, as well as next-generation materials that are better equipped to leverage mechanobiology in engineering immune niches.

Although mechanical parameters can in principle be tuned separately, they often influence one another. This has unfortunately made it quite difficult for studies to systematically dissect individual properties. For example, increasing crosslink density to raise stiffness may inadvertently reduce stress relaxation or alter pore structure [112], making it challenging to isolate which of these cues govern immune-niche formation. As such, new platform technologies that are mechanically tunable and that decouple mechanical features would significantly grow our understanding of the mechanobiology of the immune niche.

**Fig. 3:**
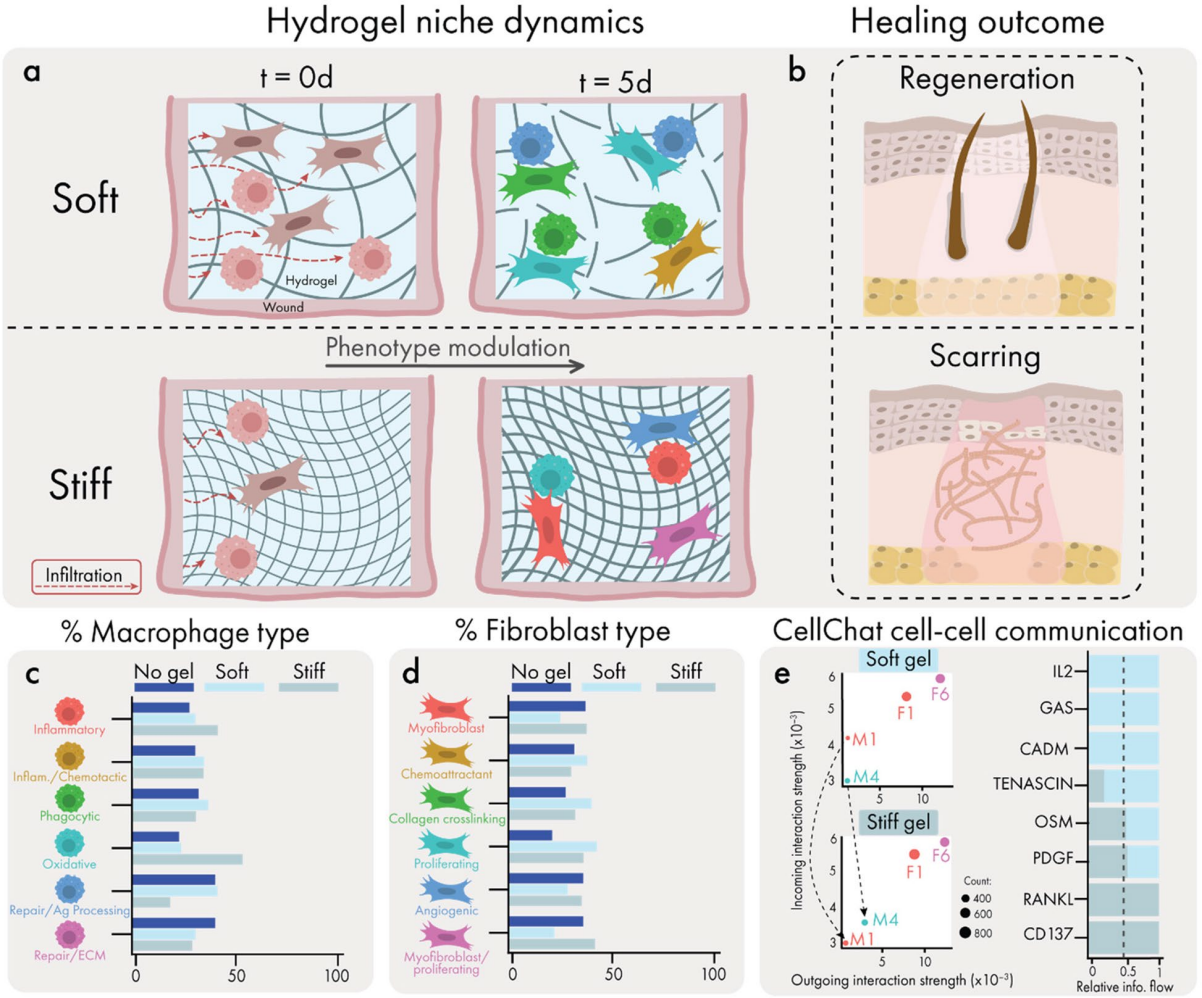
Mechanical properties regulate niche fate. **a** Schematic of immune-stromal niche dynamics in healing. Soft hydrogels support greater early cell infiltration; over time, stiffness-dependent cues bias immune and stromal cells towards distinct phenotypes. **b** Niche dynamics in soft hydrogels support regenerative repair and de novo hair follicle formation, while stiff hydrogels promote fibrotic repair and scarring. **c** Soft hydrogels are enriched in repair-associated macrophages, while stiff hydrogels show greater proportions of inflammatory and oxidation-associated fibroblasts. **d** Soft hydrogels are mostly composed of chemotaxis- and collagen maturation-associated fibroblasts, while stiff hydrogels are enriched in fibroblasts associated with scarring and contractile wound closure. **e** CellChat-inferred cell–cell communication differs by stiffness: Soft hydrogels show stronger incoming signaling to inflammatory macrophages, while stiff hydrogels show increased incoming and outgoing signaling to oxidative macrophages, with minimal differences in fibroblast signaling. Signaling molecules are also differentially enriched; stiff hydrogels upregulate fibrosis- and inflammation-associated cues while soft hydrogels upregulate signals linked to macrophage phenotype modulation. Figure adapted from Ref. [113]

Despite these challenges, existing studies have already revealed that mechanical properties can drive meaningful shifts in immune niche function, as demonstrated by Butenko et al., who used gelatin methacrylate (GelMA) hydrogels as wound dressings in a full-thickness skin injury model [113]. By varying light exposure during gelation, the authors generated (i) soft, lightly crosslinked hydrogels and (ii) stiff, highly crosslinked hydrogels with identical biochemical composition but distinct mechanical properties (Fig. 3a), allowing the effects of stiffness to be examined in isolation. Despite producing only moderate differences in overall niche composition — neutrophils roughly doubled in stiff gels (22.5% versus 9.5% in soft) while fibroblasts were somewhat more abundant in soft gels (66% versus 50% in stiff) — the two materials mediated strikingly distinct healing outcomes. Soft gels became well-integrated into the wound and supported rapid re-epithelialization and reduced scarring, whereas stiff gels remained largely unincorporated, elicited a stronger inflammatory response, and activated a foreign body response (Fig. 3b).

Intriguingly, histology revealed that soft gels were broadly infiltrated by both immune and stromal cells, while stiff gels restricted cells to the material’s periphery—likely reflecting not only increased stiffness but also the reduced pore size that accompanies higher crosslinking density, which can prevent cells from physically entering a scaffold. Because Butenko et al. used single-cell RNA sequencing (scRNA-seq) to characterize the wound niche, they were able to resolve the functional diversity of both the myeloid and stromal compartments, which together constituted the majority of cells recruited to these materials (Fig. 3c, d). Unlike lymphoid populations—where surface markers often map reliably onto many key functional subsets—myeloid and stromal cells are far more difficult to phenotype accurately using surface marker-based techniques like flow cytometry [114, 115]. Fibroblast phenotypes are even harder to define using markers alone, as their activation states are tightly linked to transcriptional programs rather than stable surface markers [116, 117]. As a result, single-cell transcriptomics offers a critical advantage by enabling simultaneous profiling of all major cellular compartments and by revealing functional states that would be difficult or impossible to identify with lower-dimensional techniques.

With this higher-resolution view, the authors found that distinct macrophage and fibroblast states emerge under different mechanical contexts in the wound niche. Their analysis identified six macrophage phenotypes and six fibroblast phenotypes, several of which shifted markedly between soft and stiff hydrogels. In soft hydrogels, macrophage states associated with phagocytosis, antigen processing, and progression toward inflammation resolution were more abundant, and fibroblast states linked to chemotaxis, proliferation, and organized matrix remodeling were similarly enriched. In stiff hydrogels, a different set of macrophage states emerged, characterized by heightened inflammatory activity, oxidative stress–related gene expression, and elevated levels of proteases involved in tissue damage [118, 119] or associated with macrophage foreign body formation [120]. Fibroblasts in these stiff-gel wounds also adopted more contractile, scar-associated phenotypes and expressed higher levels of genes associated with fibrosis. These mechanistically distinct cell transcriptional states help explain why materials with similar overall niche compositions can drive sharply different healing trajectories.

**Table:** 

**Key Technique #2: Single-Cell RNA Sequencing (scRNA-seq).** This single-cell transcriptomic technique measures RNA levels across single cells, providing a static ‘snapshot’ of genes being expressed. Cells from a tissue or matrix sample must first be isolated and dissociated into a single-cell suspension; this can be accomplished using manual dilution in a standard 96-well plate [121], microfluidic [122], or microwell [123] approaches. In droplet-based microfluidic platforms, now widely adopted in commercial systems, barcoded capture beads are used to isolate, uniquely tag, and reverse transcribe mRNA transcripts into cDNA to be sequenced using next-generation sequencing [121]. Advances in methods for isolating cells, tagging mRNA molecules, and amplifying sequences allow users to analyze the transcriptomes of ever-increasing numbers of cells [124]. Through trajectory inference methods [125], scRNA-seq snapshots from a collection of cells can be ordered along pseudotime, allowing users to capture changes in cellular states [126, 127].	

Large single-cell datasets are rich in information but can be challenging to interpret, and computational tools or additional analyses are often required to uncover deeper insights, such as how different cell populations interact [128–130]. Here, *histological observations* (rather than insights from a single-cell study) suggested that immune cells infiltrated throughout soft hydrogels, but remained largely at the periphery of stiff hydrogels. Of note, macrophages expressing *Cybb*, an oxidative marker, were found to cluster along the edge of stiff hydrogels. Furthermore, stiff hydrogels were found to be associated with fewer fibroblasts than soft hydrogels. Spatiotemporal coordination of macrophage and fibroblast signaling is known to be critical in early wound healing [131], and so these key differences in immune and stromal cell organization raise the question of how these distinct physical contexts might influence communication between the two hydrogels. To address this, the authors used CellChat, a computational method that infers potential cell–cell signaling interactions based on patterns of ligand and receptor expression [130].

CellChat analysis showed that stiff hydrogels supported communication networks enriched in inflammatory and fibrosis-associated cues, including signaling of macrophage-derived factors to myofibroblasts overexpressing contractile programs, and fibroblast-derived cues predicted to reinforce inflammatory activation in macrophages (Fig. 3e). Fibroblast to macrophage signaling was further implicated by single molecule RNA staining (RNAscope) which revealed that myofibroblasts expressing RANKL, a ligand that promotes macrophage fusion [120], were clustered at the hydrogel-dermis boundary. Although the authors did not directly measure colocalization between pro-fusogenic fibroblasts and inflammatory macrophages, each of these cells were separately determined to be associated with the periphery of stiff hydrogels, suggesting their close association. In contrast, soft hydrogels were associated with signaling pathways linked to inflammation resolution, type 2 immune polarization, matrix remodeling, and tissue repair. These findings illustrate how computational inference can reveal the communication networks at play within immune niches and provide insight into how specific macrophage and fibroblast phenotypes may regulate niche phenotype.

**Table:** 

**Key Technique #3: Cell-Cell Communication Inference Algorithms.** Computational techniques such as CellChat enable users to infer, analyze, and visualize cell-cell communication from single-cell transcriptomic data [130]. These tools use curated databases of signaling molecule interactions, including ligand-receptor interactions (multimeric ligand-receptor complexes, soluble agonists and antagonists) and membrane-bound co-receptors, to infer ligand-receptor communication and map these interactions to functional signaling pathways. CellChat begins by identifying significantly overexpressed signaling molecules through differential expression analysis, which are then used to quantify communication between two cell groups using mass-action models. The law of mass action states that the rate at which a complex is formed (here, a ligand-receptor complex) is proportional to the product of the concentration (in this context, gene expression) of the interacting molecules (ligands and receptors) [132]. Accordingly, CellChat assigns each ligand-receptor interaction a probability score based on the average expression of the ligand in a sender cell group and the corresponding receptor (and associated cofactors) in a receiver cell group. To identify biologically significant interactions, it then uses a statistical test that randomly permutes cell group labels and recalculates interaction probabilities to evaluate whether the observed communication is stronger than expected by chance [133]. CellChat summarizes these significant interactions at the pathway and network level to identify dominant signaling pathways and the key sender and receiver cell types driving communication. Finally, interactions predicted by CellChat should be validated using immunofluorescence or spatial techniques to confirm that cells are in close enough proximity to signal. Readers are referred to the following reviews for more information regarding computational tools for cell-cell communications [134] and for detailed comparisons between similar methods, including NicheNet or CellPhoneDB [135].	

Taken together, these findings show that mechanical properties influence immune niches through intertwined effects on cellular infiltration, the emergence of distinct myeloid and stromal phenotypes, and the formation of coordinated signaling networks that reinforce either reparative or inflammatory outcomes. The resulting communication networks appear to help stabilize the overall functional state of the niche, and their origins likely trace back to the initial encounters cells have as they experience mechanically distinct biomaterial environments. The availability of high-resolution transcriptomic data in the wound-healing study made it possible to identify these mechanistic links, offering a level of insight that is difficult to achieve when niche analysis is based primarily on surface-marker phenotyping, as in the vaccine studies discussed in Section “Biochemical strategies to shape immune niche composition and function”. At the same time, this system highlights a common limitation in biomaterials research that seeks to study mechanobiology: stiffness and porosity often covary within a hydrogel material system, making it challenging to determine whether cells are responding primarily to differences in elasticity, to constraints on migration, or to a combination of these cues. Developing materials that more cleanly decouple these parameters will be essential for establishing predictive rules for how mechanical environments shape immune-niche function.

### Integrin-mediated adhesion as a chemo-mechanical regulator of niche dynamics

Adhesion motifs occupy a unique position between biochemical and biomechanical regulation of immune niches. These ligands, which include short peptide sequences and other integrin-binding motifs, provide cells with specific biochemical cues that direct attachment [136]. At the same time, integrin engagement creates a physical linkage between the extracellular environment and the cytoskeleton, enabling cells to exert and sense forces [137]. Through this dual role, adhesion motifs influence how immune and stromal cells anchor, spread, apply traction, and coordinate their behavior within biomaterials [81]. As such, they represent a distinct and tunable axis of niche engineering that complements both the molecular cues described in Section “Biochemical strategies to shape immune niche composition and function” and the bulk mechanical properties discussed in Section “Mechanical properties regulate immune niche fate”.

**Fig. 4:**
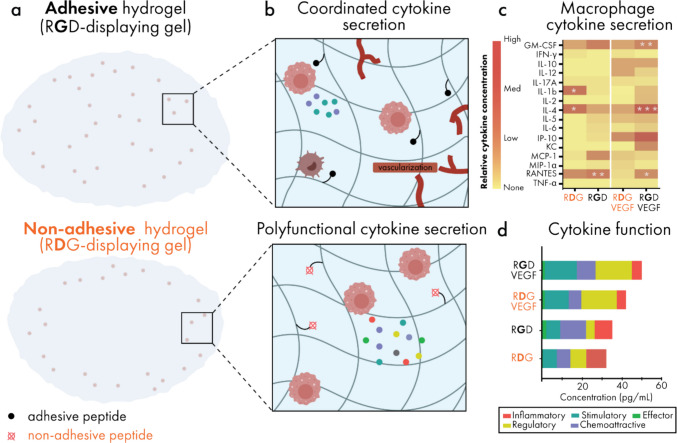
Adhesion motifs regulate immune niche functional states. **a** Hydrogels displaying peptides from adhesion domains (top) permit infiltration of immune cells, whereas hydrogels displaying non-adhesive peptides (bottom) restrict penetration of cells into hydrogels. Macrophages in each of these hydrogels are associated with unique cytokine signatures. **b** Adhesive hydrogels promote macrophage secretion of a limited number of cytokines associated with chemotactic and effector signals (top). These hydrogels are associated with increased vascularization. Non-adhesive hydrogels, on the other hand, are associated with macrophages secreting cytokines with diverse functions. **c**, **d** Inclusion of VEGF in hydrogels further promotes cytokine secretion in both RGD and RDG gels. Figure adapted from Ref. [138]

Botchwey and colleagues provide an instructive example of how adhesion motifs modulate immune-niche behavior in vivo [138]. Their system uses synthetic PEG-maleimide hydrogels that can present an integrin-binding RGD peptide or a non-binding scrambled RDG control. These hydrogels crosslink through a protease-degradable peptide and were either implanted subcutaneously or placed directly onto wounded tissue in a dorsal window chamber model. In these settings, RGD-presenting hydrogels influenced early niche behavior by accelerating the movement of myeloid cells near the material surface and increasing the number of cells in close proximity to the gel (Fig. 4a). These findings on altered motility are consistent with prior work [139], and are intuitive given the biological role of integrins.

More surprisingly, these adhesive cues also altered the local cytokine and chemokine environment. Cells associated with RGD-presenting hydrogels and the surrounding tissue produced higher levels of factors such as MCP-1, MIP-1α and β, CXCL1, and VEGF. These signals promote recruitment of monocytes, macrophages, and dendritic cells and likely contributed to the observed vascular remodeling by RGD-presenting hydrogels (Fig. 4b).

High-dimensional analyses revealed that adhesion motifs also influence the functional states of cells within hydrogels. Traditional flow cytometry detected only minor differences between RGD- and RDG-presenting materials. In contrast, single-cell proteomic profiling showed that macrophages recruited to non-adhesive hydrogels secreted highly polyfunctional mixtures of inflammatory, stimulatory, and chemoattractive cytokines. Macrophages interacting with RGD-presenting hydrogels produced fewer but more functionally similar cytokines that primarily consisted of chemotactic and effector signals, suggesting a more coordinated activation profile (Fig. 4c, d). Cytokine polyfunctionality has not been well-documented in macrophages, but in T cells, polyfunctionality is associated with enhanced antiviral [140] and anticancer [141] protection. Unbiased clustering using SPADE, a computational method that organizes single-cell data into phenotype-based clusters and maps potential transitional relationships between them [142], further identified a macrophage subset with low expression of conventional activation markers that accumulated preferentially on adhesive gels. This population exhibited distinct cytokine secretion patterns and was noted by the authors as difficult to detect through conventional gating strategies. Together, these findings indicate that adhesion motifs can alter not only how cells access and migrate around a biomaterial but also the functional programs they adopt upon interacting with its surface.

These observations indicate that there are considerable opportunities to explore how different adhesion motifs, ligand densities, or spatial patterns of ligand presentation influence immune-niche function. They also highlight the need for higher-resolution analytical approaches, since many of the phenotypic differences induced by adhesive cues are not apparent from cell-frequency measurements or traditional flow cytometry. As biomaterials are increasingly designed to present tailored combinations of adhesion ligands, understanding how these cues shape immune and stromal behavior will be essential for developing immunomodulatory materials that engage the immune system with greater precision.

## Leveraging spatial multi-omics to decipher immune niche organization and dynamics

As illustrated in Section “Mechanical properties regulate immune niche fate”, unbiased single-cell approaches like scRNA-seq address a major challenge in studying material-induced immune niches by enabling the discovery of rare cellular subtypes that lack clear profiling markers and by mapping how materials reshape their function. Yet, understanding these niches requires knowing not only which cells are present and what states they occupy, but also how they are positioned relative to one another. That is, single-cell approaches provide a detailed “parts list” of the niche, while spatial methods supply the corresponding “assembly diagram” that shows how those parts are arranged to create a functioning tissue environment. Spatial relationships such as the proximity of T cells to antigen-presenting cells, the placement of fibroblasts, or the segregation of macrophage subsets into discrete tissue zones can strongly influence how these multicellular environments coordinate to carry out a function. Without positional information, it becomes difficult to reconstruct the communication networks that shape the formation, evolution, and functional output of immune niches. Although spatial transcriptomics remains one of the most resource-intensive techniques available, its careful and targeted use in biomaterials research is already uncovering organizational principles that dissociated single-cell methods cannot resolve [143, 144].

**Table:** 

**Key Technique #4: Spatial Transcriptomics (ST).** This approach quantifies gene expression while preserving spatial information by mapping transcripts to specific locations in a tissue section [145]. Transcript capture can be performed either in a targeted manner using gene-specific probes or in a transcriptome-wide, unbiased manner, most commonly through poly(A)-based mRNA capture. A diverse range of ST modalities have been established, including region of interest-based methods, in situ imaging, and in situ sequencing approaches [146]. Among these, next-generation sequencing-based spatial barcoding platforms, such as Visium (10x Genomics), represent some of the most advanced and widely adopted technologies. These platforms differ in tissue coverage area and spatial resolution, which determine the area of tissue that can be profiled and whether transcripts are resolved at a single-cell level or aggregated across neighboring cells. The 10x Genomics Visium HD platform is among the few that achieve single-cell-scale spatial resolution using 2 x 2 µm barcoded squares while maintaining a large tissue coverage area of 6.5 x 6.5 mm [147]. Integration of ST with computational methods is critical and has enabled inferred cell-level analyses such as cell-type (Cell2location) [148] and state mapping (Starfysh) [149] from integration with scRNA-seq or histological data, identification of disease-relevant multicellular niches [149], and inference of cell-cell communication (AMICI) [150].	

**Fig. 5:**
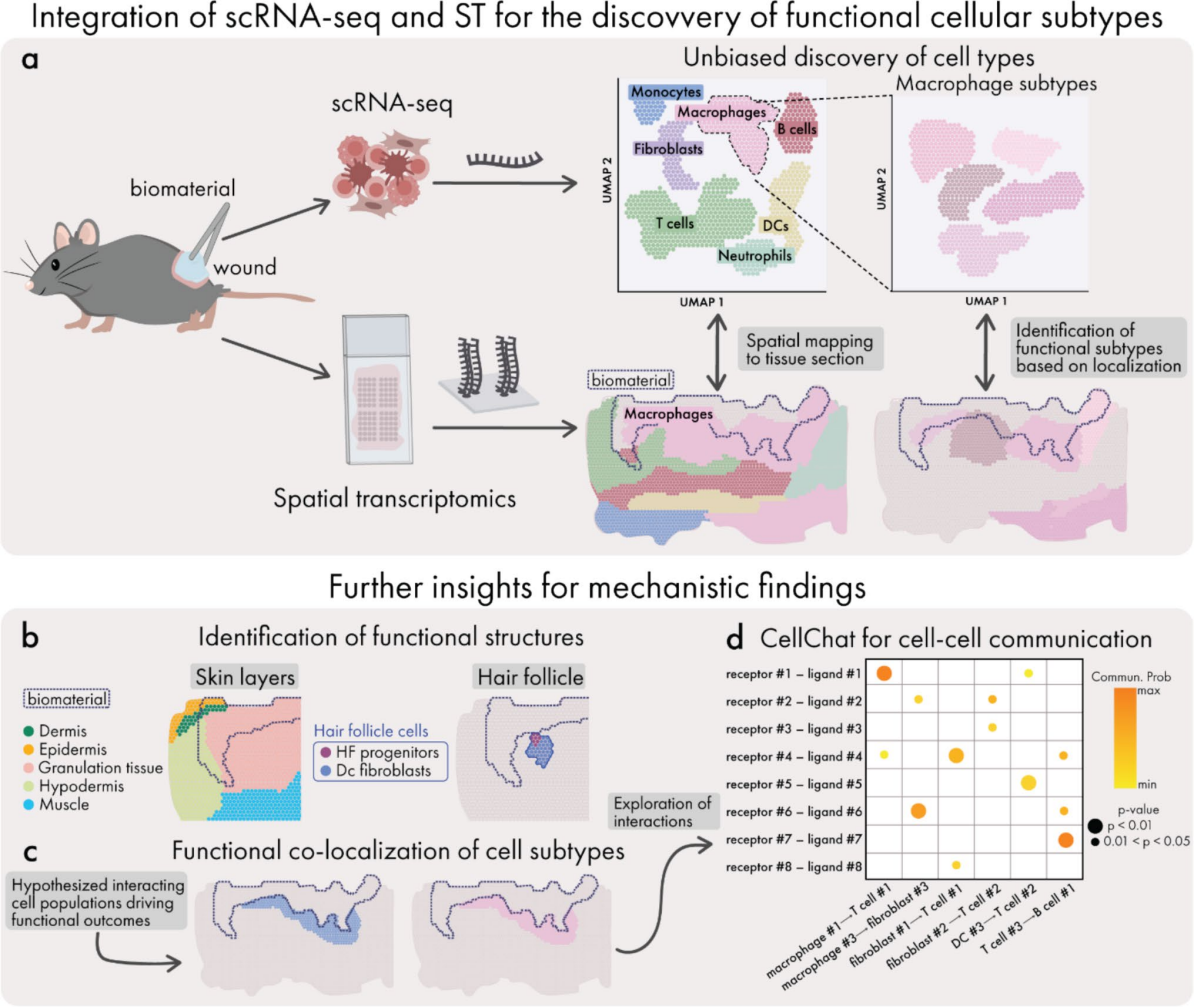
Integrating scRNA-seq and spatial transcriptomics to discover functional niche dynamics. **a** Discovering rare, functional cellular subtypes and dynamics within an immune niche benefits from the integrated use of scRNA-seq and spatial transcriptomics (ST). Unsupervised clustering of scRNA-seq data enables unbiased identification of cell subpopulations that extend beyond classical marker-based classification, including rare and previously unrecognized subtypes. Mapping these signatures into the ST tissue space then enables classification of spatially defined, functional subtypes based on their localization relative to other cells and cellular aggregates, functional structures, and implanted materials. **b** This spatial context further supports mechanistic insights by enabling the identification of organized, outcome-associated functional structures in the tissue and **c** nominating candidate driver populations through spatial co-localization within these regions. **d** Computational tools can be used to infer how cell–cell communication and signaling pathways coordinate niche dynamics to drive functional outcomes. Figure adapted from Ref. [143]

Spatial methods are particularly valuable in regenerative contexts, where the goal is to rebuild tissues with defined anatomical organization. In the skin, regeneration requires coordinated re-formation of epidermal layers, dermal fibroblast lineages, and adnexal structures such as hair follicles. To study how a biomaterial might influence this complex architecture, Yang et al. implanted a biodegradable, aligned, extracellular matrix-mimicking hydrogel beneath large full-thickness dorsal wounds and analyzed the resulting tissue using both scRNA-seq and spatial transcriptomics at multiple time points [143]. scRNA-seq identified over 35 functionally distinct cellular subpopulations, establishing a high-resolution reference that was then mapped into the spatial tissue space (Fig. 5a). The single-cell data was particularly useful for characterizing the temporal evolution of the niche, and revealed an increase in regulatory T cells, a shift toward pro-inflammatory macrophages, and expansion of regeneration-associated fibroblasts in scaffold-treated wounds. In contrast, untreated wounds were associated with greater proportions of collagen-depositing fibroblasts and type-2 macrophages during healing.

Spatial transcriptomics offered important insight into the organization of multicellular hubs around the scaffold compared to untreated wounds. In scaffold-treated wounds, papillary-fibroblasts, associated with hair-follicle neogenesis, were broadly distributed around the scaffold, whereas untreated wounds were enriched in reticular fibroblasts linked to collagen deposition and fibrotic repair (Fig. 5b). Macrophage organization followed a similar pattern: macrophages associated with early inflammatory signaling concentrated in regions surrounding the scaffold, while untreated wounds were dominated by type-2 macrophages positioned near collagen-depositing fibroblast zones. This spatial arrangement is consistent with the known functional distinctions between these macrophage states, with repair-associated macrophages supporting debris clearance, re-epithelialization, and recruitment of progenitor populations [10], whereas type-2 macrophages promote fibroblast activation and collagen cross-linking [151].

Importantly, scaffolds increased the presence of regulatory T cells, particularly around the material-dermis interface. These cells co-localized with papillary fibroblasts, suggestive of an adaptive-stromal axis aligned with regenerative trajectories. This was further supported by spatial evidence of early hair-germ-like regions at the interface between the scaffold and regenerating epidermis, marked by coordinated expression of hair-follicle progenitors and fibroblast-like dermal condensate cells. These patterns suggest that the scaffold helped create organized neighborhoods where stromal, innate, and adaptive immune populations converge in ways that favor regeneration of the complex architecture of the skin (Fig. 5c).

To understand how these cell populations coordinate their behavior, the authors used CellChat to infer ligand-receptor signaling events and then mapped these onto the spatial tissue to assess their relative positioning (Fig. 5d). In scaffold-treated wounds, regulatory T cells were the primary source of Jag1, and they showed increased communication strength with nearby pro-inflammatory macrophages through the Notch2 receptor, which was enriched around the implanted scaffold. This spatially constrained Jag1-Notch2 interaction is consistent with Notch-mediated reinforcement of M1-like over fibrosis-associated M2-like macrophage states [152]. In untreated wounds, where papillary fibroblasts and Tregs were scarce, communication was dominated by type-2 macrophages producing Tgfb1 and reticular fibroblasts expressing matching Tgfbr2 and Acvr1b receptors, activating pathways that drive collagen deposition and scar formation.

The importance of this adaptive-stromal-innate axis was underscored in mice lacking mature T cells. In these mice, scaffold-treated wounds no longer formed the Treg-papillary fibroblast niche and instead favored enrichment of collagen-depositing reticular fibroblasts and M2-like macrophages that accumulated around the scaffold. Overall, these signaling axes show that the immune niche created by the scaffold plays a central role in shaping the surrounding stromal compartment. These findings suggest that engineering materials that intentionally cultivate neighborhoods containing specific combinations of immune and stromal cells may be able to further enhance the ability of regenerative materials to recreate the architecture of complex tissues.

A related study from the same group further demonstrated how integrating scRNA-seq, ST, and deep learning approaches can be used to mechanistically resolve the multicellular dynamics that drive functional healing outcomes in material-induced niches. Here, Chen et al. used this experimental pipeline to study how age alters the cellular niche in wounds treated with Poly(lactic-co-glycolic acid) (PLGA) ECM scaffolds. They observed how dysregulated niche dynamics contribute to the attenuated functional healing response observed in aged wounds [153]. In this study, however, their focus rapidly shifted towards the myeloid compartment as they identified a specific subset of Spp1⁺Arg1⁺ macrophages that concentrated around and interacted with the scaffold (biomaterials-interacting Macrophages, Mbm). Despite comparable abundance between aged and young wounds, Mbm cells in aged wounds showed marked downregulation of gene programs involved in adaptive immune regulation, particularly helper T (Th) cell activation.

Given the interplay between adaptive immunity and wound healing observed around the scaffold, the authors further examined the T cell compartment and observed that Th cells were distributed around the scaffold at comparable proportions in young and aged wounds. In aged wounds, however, Th cells failed to upregulate Th2-associated genes consistent with the diminished type II immune responses and impaired regeneration observed in these mice. They hypothesized that this alteration arises from dysregulated Mbm-Th cell interactions that fail to drive Th cell differentiation into Th2 cells, potentially linked to Mbm cell senescence. Using CellChat, they showed that Mbms in young mice exhibited greater outgoing signaling through pathways linked to immune activation, whereas aged Mbm displayed reduced signaling capacity overall, aligning with their loss of adaptive immune-regulating programs and increased senescence.

This collective disruption indicates that, although aged wounds retain similar cellular compositions and spatial organization, they lose the functional transcriptional programming and signaling environment that leads to regenerative healing outcomes in young mice. In addition to highlighting a critical variable of the host age in the formation of biomaterials-associated immune niches, the study illustrates how jointly resolving cellular composition, spatial organization, and cell–cell communication is essential for interpreting the functional behavior of biomaterial-induced niches.

This concept is further supported by work from by Elisseeff and coworkers, who examined biomaterials-mediated tissue repair responses in aging mice [154]. Using scRNA-seq, histology, and cell–cell communication inference, Han *et al.* observed skewed immune phenotypes in draining lymph nodes and dysregulation of T cell–stromal interactions within the injury niche. Senescent fibroblasts induced Th17 differentiation of T cells from aged mice, but not young mice. Additionally, sequencing analysis and flow cytometry revealed a systemic type 3 immune response in aged mice that impaired biomaterials-mediated regeneration. Administration of IL17-neutralizing antibodies alongside regenerative biomaterials was partially effective in improving age-associated healing. These studies, and others by these groups, have been fundamental in establishing that host-intrinsic factors—particularly aging—are critical determinants of immune niche composition and function, shaping both local cellular organization and systemic immune tone in ways that directly influence material performance. Importantly, reaching these conclusions required the integration of multi-omic and spatial profiling approaches that remain uncommon in biomaterials research, illustrating the analytical depth needed to resolve how host biology intersects with material-driven niche dynamics.

Together, these studies demonstrate how spatial transcriptomics can reveal organizational and communication patterns that are difficult to resolve with dissociated single-cell methods alone. Extracting these insights often requires close collaboration with computational experts who can manage large spatial datasets, integrate transcriptional and positional information, and infer biologically meaningful interaction networks. As these approaches become more widely applied, they are poised to give biomaterials engineers a clearer understanding of the spatial relationships and signaling partnerships that define an effective immune niche. We encourage those in the field to think not only about which immune or stromal cells a material should recruit, but also about how to intentionally cultivate their spatial arrangement. Achieving this level of control will likely require new innovations in material patterning and the spatially controlled presentation of biochemical and mechanical cues.

## Future outlook: opportunities for interdisciplinary collaboration at the intersection of biomedical engineering, materials science and systems immunology

The clinical translation of injectable hydrogels is advancing rapidly, with a growing number of systems entering clinical research across a range of indications [155, 156]. This progress has brought important regulatory and practical considerations — including injectability, biocompatibility, degradation, route of administration, complexity, cell-material crosstalk, and cell recruitment — into sharper focus, and we refer readers to recent reviews for a comprehensive treatment of these topics [5, 157]. As immune engineering applications continue to mature, it is becoming clear that achieving predictable and durable outcomes will require more than controlling which signals a material delivers — it will require understanding how those signals orchestrate the formation and function of multicellular communities at the implant site. Yet our grasp of how immune niches take hold, evolve, and ultimately determine material performance remains nascent. We are at an early and exciting moment in which the tools to ask these questions rigorously are only just becoming available to biomaterials researchers.

The studies summarized here highlight several central questions will shape the next phase of immune niche engineering. One important challenge is to understand the plasticity of immune niches; how do their cellular composition and functional states evolve over time in response to biomaterials? Related to this is the question of which features of a niche are most predictive of outcome: what is the relative importance of the identity of the cells present, the transcriptional programs they adopt, or the signaling relationships they form with neighboring stromal and immune populations? The studies we discussed described the influence of biochemical, mechanical, and environmental cues in isolation, but how do these signals work together to determine cell fate? There is also a need to define the mechanisms that drive divergent outcomes. Addressing these questions will require temporal and functional studies that capture the dynamic progression from material implantation to niche formation and, ultimately, to niche function.

Technical advances will play a major role in answering these questions. High-dimensional analyses, including multiparametric flow cytometry, single-cell transcriptomics, spatial transcriptomics, and computational inference of cellular communication networks, are beginning to reveal how immune and stromal cells coordinate within biomaterial environments. However, realizing the full potential of these approaches will require true interdisciplinary collaboration. Computational scientists and systems biologists bring the analytical depth that multi-omics datasets demand, but applying these techniques to biomaterials introduces sample preparation challenges that differ substantially from processing native tissues—challenges that cannot be navigated without deep materials expertise. Conversely, biomaterials researchers seeking to deploy these tools will need partners who can implement them rigorously. Meaningful progress will therefore require both communities working together, alongside additional tools such as in vivo lineage tracing, long-term fate mapping of material-conditioned cells, and multimodal analyses that combine spatial, temporal, and functional readouts.

There is also a major opportunity for machine learning and multimodal language models to assist in the design and generation of biomedically relevant materials. Machine learning has been applied to the prediction of polymer properties from a given structure, or the prediction of polymer structures with desired properties, but applications in biomaterials design have been limited thus far [158, 159]. A key obstacle is the lack of robust standards across experimental methods, material characterization, biological reagents, and cell lines, which limits the size and consistency of datasets these models can draw on. Overcoming these obstacles will be essential to integrating computational tools meaningfully into biomaterials research. As these methods become more accessible, deploying them strategically in contexts where coordinated multicellular mechanisms are most likely to influence material performance will be essential.

At the same time, new opportunities are emerging for material design. A near-term priority will be the development of platforms that can more cleanly decouple the biochemical, mechanical, and adhesive parameters discussed here, enabling their systematic and independent study. Building on that foundation, the longer-term goal is to develop biomaterials that intentionally cultivate immune-stromal architectures—for example by establishing local biochemical gradients, or by patterning materials to bring particular fibroblast, macrophage, or T cell subsets into productive proximity. Achieving this level of spatial and functional control will require innovations in both materials chemistry and fabrication methods.

Equally important will be a deeper understanding of the host contribution to immune niche formation. The tissue site of implantation is itself a critical variable, as different anatomical locations harbor distinct resident immune populations and differ in their degree of vascularization and lymphatic drainage, all of which shape cellular infiltration and niche composition [160]. Beyond location, the physiological state of the host—including age, metabolic health, and pre-existing inflammatory conditions—will influence how niches form and function [154]. High glycolytic activity by implant-associated macrophages, for instance, has been linked to fibrosis and chronic inflammation, and metabolic dysregulation at the systemic level may bias resident and recruited cells toward particular immune programs before a material is ever implanted [161, 162]. Understanding how these host-intrinsic factors interact with material design parameters will be essential for predicting niche outcomes across diverse patient populations.

Designing materials that meet these challenges will require close collaboration across disciplines and continued innovation in both analytical and fabrication approaches. Although much remains to be learned, this interdisciplinary research trajectory has remarkable potential to produce increasingly functional biomaterials that reprogram and harness the immune system to improve human health.

## Data Availability

Not applicable (no new data generated).
